# Growing up in poverty, growing old in frailty: the life course shaping of health in the United States, England and Europe—a prospective and retrospective study

**DOI:** 10.1038/s41598-025-99929-2

**Published:** 2025-05-03

**Authors:** Gindo Tampubolon

**Affiliations:** 1 UK NIHR Policy Research Unit on Healthy Ageing, Manchester, UK; 2 Global Development Institute, University of Manchester, Manchester, UK

**Keywords:** U.S., England, Europe, Childhood poverty, Frailty, Life course, Healthy ageing, Fixed effects, Random effects, Risk factors, Public health

## Abstract

Childhood poverty is directly associated with many health outcomes in late life irrespective of youth health and of health system variation. The childhood poor in the United States (U.S.), England and Europe have reported worse cognitive, muscle and mental functions in their fifties to nineties. But it is not known whether they have higher probabilities of experiencing frailty as their childhood recollections are likely to be erroneous. Nearly 80,000 adults aged 50 and older retrospectively recalled their childhood conditions around ten and underwent prospective examinations to construct their Fried’s frailty phenotype. Childhood conditions in England and Europe included number of books, number of rooms, number of people, presence of running hot or cold water, fixed bath, indoor lavatory and central heating (English Longitudinal Study of Ageing; Survey of Health, Ageing and Retirement in Europe). In the U.S., these were mostly replaced with financial hardship indicators including having to move because of family debt (Health and Retirement Study). Childhood poverty is a latent construct of error-laced recollection and its distal association with frailty phenotype was estimated with a fixed effects probit model. Sensitivity analyses were conducted using a random effects model and stratifying on sex. Childhood poverty associates with higher probabilities of being frail (0.1097 ± 0.0169, p < 0.001) in 29 countries including U.S., England and Europe. Furthermore, women have higher probabilities of being frail (0.3051 ± 0.0152, p < 0.001). Age, education, wealth, marital status and youth illness exert influences on the probabilities of being frail. Evidence is mounting that childhood can last a life time, affecting cognitive muscle and mental functions, and now frailty. This evidence calls for urgent actions to eliminate child poverty on account of its lifelong rewards.

## Introduction

Childhood poverty is a risk factor for old age disability, dysfunction and disease in the United States (U.S.), England, China and Europe^1–8^. This raises the question of whether childhood poverty also marks older adults’ frailty, a syndrome of systemic decline across multiple organ functions which often entails adverse clinical outcomes, high costs of care and mortality^9,10^. This question extends the term ‘long arm of childhood conditions,’ coined by Hayward and Gorman who studied the influence of childhood poverty on mortality in American males in their forties and fifties^11^. Large and ongoing ageing surveys have provided evidence to support the links across the life course. Twenty eight trans-Atlantic countries from U.S. through England to Israel report life course links that persist, marking muscle function, depression status and cognitive function of older adults who grew up in poverty^3^. With such cross-country comparative design, any new evidence on life course shaping of frailty can be used to promote efforts during the UN Decade of Healthy Ageing^12^. Such efforts are urgent because frailty—typically though not invariably measured as frailty phenotype or index—reduces older adults’ wellbeing while imposing high costs on society^9,13,14^.

This work aims to test the hypothesis of life course shaping of frailty in U.S., England and Europe which posits that childhood poverty continues to mark old age frailty even after youth or adult conditions are considered. The lifelong association between poverty and frailty remains. A recent test of the hypothesis was applied to sarcopenia, a narrower geriatric syndrome than frailty which captures *multiorgan* dysfunction^3^. To achieve the aim three sister studies (Health & Retirement Study—HRS, English Longitudinal Study of Ageing—ELSA, Survey of Health Ageing & Retirement in Europe—SHARE)^15–17^ covering 29 countries are used in a cross-country fixed effect design following earlier studies^3,4^. To guide further steps the lens of social determinants of health as links across the life course is adopted, defined as “conditions in the environments in which people are born and *raised*, live, learn, work, play, worship, and *age* that affect a wide range of health, functioning, and quality-of-life outcomes”^18,19^. The investigation which posited the life course association studied midlife U.S. males; it was silent on the experience of large sections of the population i.e. females and adults living much longer. Thus cross-country studies of both sexes constitute a specific test of the life course shaping of frailty, helping to fathom its reach and limits. In sum, how one was raised materially in the U.S., England and Europe can have lasting effects on the chance of experiencing frailty in old age.

When designing the test one encounters different concepts and indicators for childhood conditions in the empirical literature. Childhood poverty, childhood socioeconomic position and adverse childhood experience were some examples. This study does not use the adverse childhood experience scale for two reasons. First, the scale is complementary to the other concepts. Second, the scale has more psychological or subjective elements which suggests more susceptibility to bias. I am not aware of any test of the magnitude of the bias, for instance by comparing an independent report during childhood and during old age, unlike childhood poverty which has undergone such a test^1,2^.

## Materials

### Outcome: frailty phenotype

The analytic sample was constructed from a family of ageing studies: HRS, ELSA and SHARE^15–17^. Already in 2015 Theou and colleagues found that there were 226 constructs of frailty^20^. I avoided adding another construct, because replicability is a critical practice for cross-country study, choosing to follow closely studies of Fried, Bandeen-Roche and colleagues for HRS^21,22^, Leme and de Oliveira for ELSA^23^, and Santos-Eggimann and colleagues’ for SHARE^24^. Please consult these for definitive details. Briefly presented in Table 1, when meeting at least three out of five indicators the person is considered frail, while meeting zero to two indicators is considered non-frail; altogether making frailty a binary outcome following a recent study^23^. By following previous studies, replicability as a good practice is enhanced. The indicators are exhaustion, unexpected weight loss, weakness, low energy and slowness.

**Table 1:** Indicators and variables of frailty phenotype in HRS, ELSA and SHARE^21–25^. *CESD* Center for Epidemiologic Study Depression scale.

Indicators	HRS	ELSA	SHARE
Period	Current, 2020/21	Current, 2020/21	Current, 2020/21
Exhaustion	CESD item	CESD item	Self-report
Weight loss	Body mass index	Body mass index	Self-report of appetite
Weakness	Grip strength	Grip strength	Grip strength
Low energy	Self-report of physical activity	Self-report of physical activity	Self-report of physical activity
Slowness	Gait speed	Gait speed	Self-report of two mobility problems

### Childhood information

Participants were asked retrospectively about their childhood conditions up to eight decades earlier, then prospectively about their health. Measures of childhood poverty listed below are based on retrospective reports of adults aged 50–95 (average 66), raising concern about recall error. For example, in 2008 some 2500 Britons aged fifty were asked to recall the numbers of rooms and people in their homes when they were eleven. Only one in three got both numbers right^1,2^. Here the participants have an average age of 66. It is scarcely plausible that those sixteen years older than fifty achieved perfect recall. Thus studies have responded to the concern of recall error by devising latent constructs for childhood poverty^1–7^. Following these, the latent class of childhood poverty, derived from sets of childhood condition variables, is used as the key risk factor in old age health.

The empirical literature on cross-country comparison offers another building block to enable comparison of concepts despite less than exact equivalence of variables e.g. on depression between U.S. and Europe as well as on allostatic load between U.S. and England^26,27^. ELSA collected life history in wave 3, while SHARE collected life history in wave 3 (and 7 for those who did not provide it the first time). The ELSA and SHARE variables have been described elsewhere^2,3,28,29^. HRS collected childhood information in the core interview at each wave but, crucially, this is not closely comparable with variables from the other side of the Atlantic, where instead the survey opted to elaborate on financial situation. This information is augmented with the ad hoc Life History Mail Survey 2017. Such variables were also used in the precursor of SHARE, the Survey of Income and Living Conditions in Europe which has been studied with the same method here^1^. Although ageing studies in U.S., England and Europe have close family resemblance, the differences are worth listing. Table 2 lists variables collected in each study, noting the emphasis in HRS on financial information, used to derive latent class of childhood poverty in previous studies^2,3,28,29^.

**Table 2:** Retrospective childhood variables in HRS, ELSA and SHARE.

Country	HRS	ELSA	SHARE
Period variable	Childhood	Childhood	Childhood
	Number of rooms	Number of rooms	Number of rooms
	Number of people	Number of people	Number of people
	Number of books	Number of books	Number of books
	Ever experienced:	Physical facilities:	Physical facilities:
	being financially poor, had to move or be helped because of financial difficulty, had to live with grandparents, father unemployed	indoor toilet, hot and cold running water, central heating, fixed bath	indoor toilet, hot and cold running water, central heating, fixed bath

## Methods

To test the life course shaping hypothesis, the latent class of poverty in childhood period was derived following previous studies of HRS, ELSA and SHARE^1–3^. It is then matched with current period frailty (2020/21). For modelling associations, a set of confounders is included as standard in the literature including father/parent’s occupation (childhood period), youth illness (youth period) and current period variables including age, sex, education, marital status, wealth, ethnicity and country^2,5,30,31^. Following the literature, fixed effects probit model was estimated and standard error correction was applied because the key exposure of childhood poverty is an estimated latent class^3,32^. Random effects probit model and stratification by sex are conducted as parts of sensitivity analyses. Latent GOLD syntax version 6 was used for all modelling and no correction for multiple comparison was applied because there is only one outcome. All statistical tests (*t* and chi-squared) were done with Stata version 18 (College Station, Texas, U.S.) and statistical significance was set at 5%^33^.

## Results

### Missing data or non-response for retrospective interviews

I begin by describing the analytic sample which comprises prospective sample members who agreed to retrospective interviews. Not all of them did and there may be significant differences between the analytic *vs* declined sample, tested using ${\chi }^{2}$ for binary variable and *t* tests for continuous variable (Table 3). In the HRS the analytic sample has 61% females and 39% males, with ${\chi }_{1}^{2}=15.7$ and $p {\text{value}}<0.001.$ In contrast, both in SHARE and ELSA analytic samples females are no more likely to agree to retrospective interviews. Meanwhile in the HRS the analytic sample has significantly older members while in SHARE and ELSA the opposite is true.

**Table 3:** Missing data or non-response patterns across analytic *vs* declined sample in HRS, SHARE and ELSA.

Survey	Variable	Declined sample	Analytic sample
HRS	Female	58%	61%
	Male	42%	39%
		${\chi }_{1}^{2}=15.7$	$p<0.001$
	Mean age	64.6 year	68.8 year
			$$p<0.001$$
ELSA	Female	56%	55%
	Male	44%	45%
		${\chi }_{1}^{2}=2.0$	$p=0.160$
	Mean age	65.1 year	64.4 year
			$$p=0.022$$
SHARE	Female	56%	58%
	Male	44%	42%
		${\chi }_{1}^{2}=1.9$	$p=0.165$
	Mean age	72.2 year	67.1 year
			$$p<0.001$$

Table 4 collects features of the analytic sample which has 57% female, mean age 66.3 year and one in five was childhood poor. Times ill in youth is non-negligible.

**Table 4:** Summary features of the analytic sample with categorical variables in numbers (percentages) and continuous variables in means (standard deviations). Sources HRS, ELSA and SHARE (the last does not release ethnicity variable).

	Male	Female	Total
HRS (U.S.)	N = 8504	N = 11,656	N = 20,160
Child			
Non-poor	7952 (93.5%)	10,996 (94.3%)	18,948 (94.0%)
Poor	552 (6.5%)	660 (5.7%)	1212 (6.0%)
Frailty phenotype			
Normal and pre-frail	8349 (98.2%)	11,476 (98.5%)	19,825 (98.3%)
Frail	155 (1.8%)	180 (1.5%)	335 (1.7%)
Age	66.1 (10.9)	66.8 (11.4)	66.5 (11.2)
Times ill (youth)	0.1 (0.3)	0.1 (0.3)	0.1 (0.3)
College			
High school or lower	7504 (88.2%)	10,403 (89.3%)	17,907 (88.8%)
College	1000 (11.8%)	1253 (10.7%)	2253 (11.2%)
Marital status			
Single, never married	661 (7.8%)	885 (7.6%)	1546 (7.7%)
Married/union	223 (2.6%)	284 (2.4%)	507 (2.5%)
Widowed, separated	7620 (89.6%)	10,487 (90.0%)	18,107 (89.8%)
Father’s job: manual occ			
False	7382 (86.8%)	10,392 (89.2%)	17,774 (88.2%)
True	1122 (13.2%)	1264 (10.8%)	2386 (11.8%)
Bottom quintile			
False	7328 (86.2%)	9449 (81.1%)	16,777 (83.2%)
True	1176 (13.8%)	2207 (18.9%)	3383 (16.8%)
Race/ethnicity			
No	2825 (33.2%)	3909 (33.5%)	6734 (33.4%)
White/Caucasian	5679 (66.8%)	7747 (66.5%)	13,426 (66.6%)
ELSA (England)	N = 3261	N = 4258	N = 7519
Child			
Non-poor	2663 (81.7%)	3455 (81.1%)	6118 (81.4%)
Poor	598 (18.3%)	803 (18.9%)	1401 (18.6%)
Frailty phenotype			
Normal and pre-frail	3124 (95.8%)	4073 (95.7%)	7197 (95.7%)
Frail	137 (4.2%)	185 (4.3%)	322 (4.3%)
Age	64.8 (10.3)	64.4 (11.4)	64.5 (10.9)
Times ill (youth)	0.5 (0.9)	0.6 (1.1)	0.6 (1.0)
College			
High school or lower	2619 (80.3%)	3759 (88.3%)	6378 (84.8%)
College	642 (19.7%)	499 (11.7%)	1141 (15.2%)
Marital status			
Single, never married	214 (6.6%)	214 (5.0%)	428 (5.7%)
Married/Union	2448 (75.1%)	2599 (61.0%)	5047 (67.1%)
Widowed, separated	599 (18.4%)	1445 (33.9%)	2044 (27.2%)
Father’s job: manual occ			
False	3041 (93.3%)	3990 (93.7%)	7031 (93.5%)
True	220 (6.7%)	268 (6.3%)	488 (6.5%)
Bottom quintile			
False	2668 (81.8%)	3369 (79.1%)	6037 (80.3%)
True	593 (18.2%)	889 (20.9%)	1482 (19.7%)
Race/ethnicity			
No	77 (2.4%)	102 (2.4%)	179 (2.4%)
White/Caucasian	3184 (97.6%)	4156 (97.6%)	7340 (97.6%)
SHARE (Europe)	N = 22,049	N = 29,700	N = 51,749
Child			
Non-poor	16,631 (75.4%)	21,850 (73.6%)	38,481 (74.4%)
Poor	5418 (24.6%)	7850 (26.4%)	13,268 (25.6%)
Frailty phenotype			
Normal and pre-frail	20,109 (91.2%)	24,716 (83.2%)	44,825 (86.6%)
Frail	1940 (8.8%)	4984 (16.8%)	6924 (13.4%)
Age	66.7 (9.0)	66.2 (9.9)	66.4 (9.6)
Times ill (youth)	0.2 (0.6)	0.3 (0.7)	0.3 (0.7)
College			
High school or lower	16,830 (76.3%)	23,573 (79.4%)	40,403 (78.1%)
College	5219 (23.7%)	6127 (20.6%)	11,346 (21.9%)
Marital status			
Single, never married	1240 (5.6%)	1343 (4.5%)	2583 (5.0%)
Married/Union	17,773 (80.6%)	19,116 (64.4%)	36,889 (71.3%)
Widowed, separated	3036 (13.8%)	9241 (31.1%)	12,277 (23.7%)
Father’s job: manual occ			
False	16,963 (76.9%)	22,520 (75.8%)	39,483 (76.3%)
True	5086 (23.1%)	7180 (24.2%)	12,266 (23.7%)
Bottom quintile			
False	18,637 (84.5%)	23,290 (78.4%)	41,927 (81.0%)
True	3412 (15.5%)	6410 (21.6%)	9822 (19.0%)

The fixed effects probit model coefficients explaining frailty are collected in Table 5 which shows that the childhood poor report significantly higher probabilities of frailty (0.1097 ± 0.0169, *p* value ≤ 0.0001). In the U.S., England and Europe growing up in poverty goes with growing old in frailty. Females report higher probability of being frail (0.3051, *p* ≤ 0.0001) and the probability increases as people age (0.0398, *p* ≤ 0.0001). Briefly, the other covariates suggest that college education (compared to high school or lower) associates with lower risks of being frail (− 0.2540, *p* ≤ 0.0001). Importantly, reflecting the social determinants framework above, youth illness is significant and in the expected direction (0.2334, *p* ≤ 0.0001). Being in the bottom third of wealth distribution associates with higher risks of frailty, as does having a father with manual or elementary occupation. This warrants interpreting childhood poverty coefficient as a fully adjusted association.

**Table 5:** Probit coefficients explaining frailty in HRS, ELSA and SHARE. Constant includes the reference country England.

Variable	Coefficient	Standard error	90% conf	interval	*P* value
Constant	− 5.2006	0.0700	− 5.3378	− 5.0634	≤ 0.0001
Childhood poor	0.1097	0.0169	0.0766	0.1428	≤ 0.0001
Sex, Female	0.3051	0.0152	0.2753	0.3349	≤ 0.0001
Age	0.0398	0.0008	0.0382	0.0414	≤ 0.0001
College	− 0.2540	0.0212	− 0.2956	− 0.2124	≤ 0.0001
Single, never married	0.0098	0.0353	− 0.0594	0.0790	0.78
Married/union	− 0.0914	0.0174	− 0.1255	− 0.0573	≤ 0.0001
Youth illness	0.2334	0.0090	0.2158	0.2510	≤ 0.0001
Father had manual occ	0.0346	0.0180	− 0.0007	0.0699	0.055
Bottom wealth tertile	0.1816	0.0203	0.1418	0.2214	≤ 0.0001
Country ref: England					
Austria	1.0666	0.0463	0.9759	1.1573	≤ 0.0001
Germany	0.9874	0.0425	0.9041	1.0707	≤ 0.0001
Sweden	0.7525	0.0465	0.6614	0.8436	≤ 0.0001
Netherlands	0.8745	0.0567	0.7634	0.9856	≤ 0.0001
Spain	1.2681	0.0401	1.1895	1.3467	≤ 0.0001
Italy	1.3377	0.0402	1.2589	1.4165	≤ 0.0001
France	1.1892	0.0398	1.1112	1.2672	≤ 0.0001
Denmark	0.8807	0.0475	0.7876	0.9738	≤ 0.0001
Greece	1.3729	0.0614	1.2526	1.4932	≤ 0.0001
Switzerland	0.6484	0.0530	0.5445	0.7523	≤ 0.0001
Belgium	1.1513	0.0407	1.0715	1.2311	≤ 0.0001
Israel	1.4092	0.0588	1.2940	1.5244	≤ 0.0001
Czechia	0.9814	0.0423	0.8985	1.0643	≤ 0.0001
Poland	1.3533	0.0435	1.2680	1.4386	≤ 0.0001
Luxembourg	0.8424	0.0753	0.6948	0.9900	≤ 0.0001
Hungary	1.3522	0.0653	1.2242	1.4802	≤ 0.0001
Slovenia	0.9299	0.0460	0.8397	1.0201	≤ 0.0001
Estonia	1.1239	0.0410	1.0435	1.2043	≤ 0.0001
Croatia	1.1975	0.0565	1.0868	1.3082	≤ 0.0001
Lithuania	1.1440	0.0547	1.0368	1.2512	≤ 0.0001
Bulgaria	1.3211	0.0594	1.2047	1.4375	≤ 0.0001
Cyprus	1.2528	0.0720	1.1117	1.3939	≤ 0.0001
Finland	0.7931	0.0687	0.6584	0.9278	≤ 0.0001
Latvia	0.9777	0.0699	0.8407	1.1147	≤ 0.0001
Malta	1.1812	0.0668	1.0503	1.3121	≤ 0.0001
Romania	1.3181	0.0554	1.2095	1.4267	≤ 0.0001
Slovakia	1.5224	0.0591	1.4066	1.6382	≤ 0.0001
U.S	0.3587	0.0404	0.2795	0.4379	≤ 0.0001

**Fig. 1:**
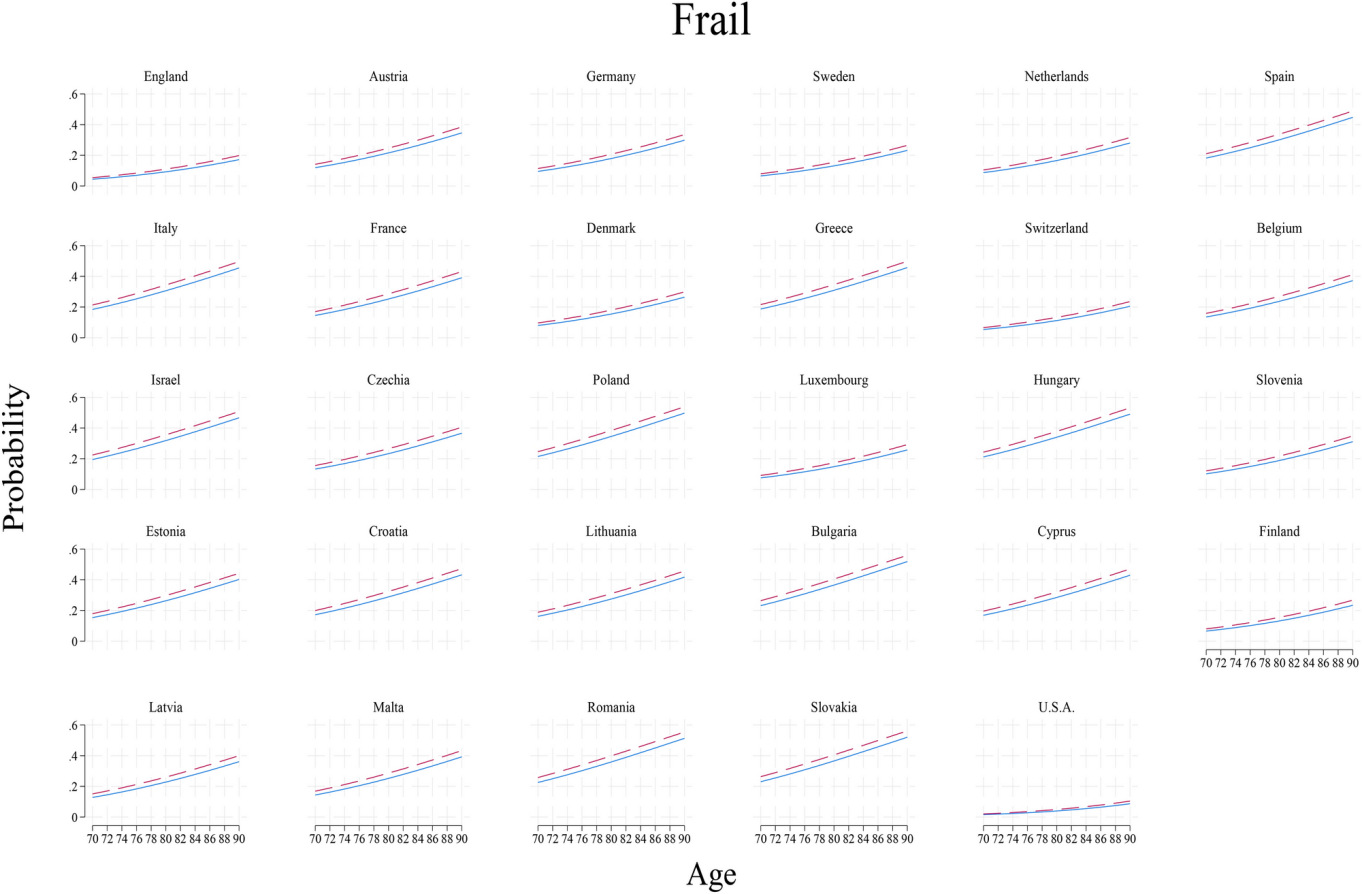
Probabilities of Frail among the childhood poor (dash) and non-poor (solid) in older people aged 70–90 years in U.S., England and Europe based on models in Table 3 where all covariates are set at the sample averages. Analysis of HRS, ELSA and SHARE.

To bring this forward, for each of the 29 countries the predicted probabilities of frailty between age 70 and 90 are plotted and distinguished by childhood poverty. This shows at a glance how childhood poverty shapes frailty in old age across a wide range of history and health systems of rich countries (Fig. 1).

The trellis plot reveals several key patterns. First, age is an important risk factor though it works differently in different countries, validating the fixed effect design. For example, compare the levels and slopes of Slovakia and U.S. (the last two panes). Second, childhood poverty puts people at a disadvantage in old age, putting the dashed lines always above the solid lines. This is statistically significant as shown in Table 5. Third, the regional patterns of Europe are pronounced (Northern, Southern and Eastern Europe). This can be seen with Sweden, Finland and Denmark as one group, Spain, Italy and Greece as another, and Croatia, Romania and Slovakia as yet another. Together, the variation is marked, preventing one single summary pattern from representing all high income countries.

To check the robustness of these results, two sensitivity analyses were conducted replacing the fixed effect design with a random effect one, effectively taking these countries as randomly drawn from a population of rich countries, then stratifying by sex. The results above stand (Tables 6 and 7).

**Table 6:** Sensitivity analysis: Random effects probit coefficients explaining frailty in U.S. (HRS), England (ELSA) and Europe (SHARE).

Variable	Coefficient	Standard error	P value
Const	− 4.1054	0.2494	≤ 0.0001
Childhood poor	0.1509	0.0244	≤ 0.0001
Sex, Female	0.3068	0.0620	≤ 0.0001
Age	0.0406	0.0032	≤ 0.0001
College	− 0.2609	0.0242	≤ 0.0001
Single, never married	0.0139	0.0290	0.63
Married/union	− 0.0848	0.0223	≤ 0.0001
Youth illness	0.2386	0.0114	≤ 0.0001
Father had manual occ	0.0483	0.0298	0.10
Bottom wealth tertile	0.1886	0.0559	0.0007
Random effects variance	0.0989	0.0113	≤ 0.0001

**Table 7:** Sensitivity analysis: Probit coefficients with random effects and stratified by sex, explaining frailty in U.S. (HRS), England (ELSA) and Europe (SHARE).

Variable	Coeff	Female	P value	Coeff	Male	P value
		Standard error			Standard error	
Constant	− 4.2401	0.2096	≤ 0.0001	− 4.0343	0.1974	≤ 0.0001
Childhood poor	0.1830	0.0405	≤ 0.0001	0.1443	0.0294	≤ 0.0001
Age	0.0423	0.0030	≤ 0.0001	0.0385	0.0031	≤ 0.0001
College	− 0.2479	0.0269	≤ 0.0001	− 0.2828	0.0421	≤ 0.0001
Single, never married	− 0.0057	0.0400	0.89	0.0580	0.0500	0.25
Married/union	− 0.0590	0.0238	0.013	− 0.0704	0.0278	0.011
Youth illness	0.2449	0.0114	≤ 0.0001	0.2314	0.0165	≤ 0.0001
Father had manual occ	0.0573	0.0316	0.069	0.0108	0.0359	0.76
Bottom wealth tertile	0.2071	0.0261	≤ 0.0001	0.1913	0.0448	≤ 0.0001
Random effects var	0.0736	0.0067	≤ 0.0001	0.1	0.0090	≤ 0.0001

## Discussion and conclusion

Compelling evidence is accumulating that the life course shaping of health reaches from childhood into old age. Childhood poverty continues to shape the health of older people around the world even in rich nations across wide outcomes of ageing including frailty. This is the first evidence in 29 nationwide representative populations of how childhood poverty plays a role in frailty variations in older adults. This operates in diverse health systems: in a national health system (England), a largely private health system (U.S.) and everything in between (27 European countries). The UK’s NHS is more than 75 years old and has accompanied Britons in the study cohorts throughout their life courses. Using latent construct to recover child poverty status from sets of error-laced indicators, the analysis shows important associations between childhood poverty and frailty that are consistently observed across 29 rich countries enhancing our appreciation of the life course shaping of health in old age.

What mechanism lies behind this shaping is an important question. I have tried to offer an epigenetic explanation and evidence for the role of childhood poverty as a risk factor, showing that in U.S. the childhood poor aged epigenetically faster^3^. Poverty in childhood is shown to induce epigenetic change through increasing methylation rates which leaves a mark that lasts until old age^3,34,35^. That childhood lasts a lifetime, we knew. Now we know it also shapes the probability of experiencing the frailty syndrome.

### Reflection on recent literature

These findings echo recent studies in different countries, though not all^1–3,5–7,28,29^. While consensus has not yet arrived, the evidence is largely supportive of a life course shaping of frailty. The contrast with the new findings here can arise for many reasons below. Here nationally representative samples are used, comparable across 29 countries. Further, fixed effects capturing idiosyncratic country effects such as unique health system and history, are included. All agree in showing that the childhood poor have more frailty in old age.

But a few studies on the life course shaping of health in older ages have reported different results, which may be due to variations in variable construction, outcomes, methods, health systems and broader structures of societies. Vable and coauthors in U.S. suggest that once socioeconomic mobility in adulthood is considered, no direct association remains between childhood economic status and old age health^7^. On this side of the Atlantic, studies in Sweden and Europe have also shown varying associations between poverty or economic status in childhood and old age health^4–6^. But the constructs of childhood conditions vary. Lennartsson and colleagues in Sweden depart from the usual practice of ignoring recall bias by using a latent construct of childhood poverty, in fact a full structural model to explain old age health indicated by pain, fatigue and breathing difficulty; meanwhile Pakpahan and colleagues also used a structural model to explain self-rated health in old age in 13 European countries^4,5^.

In Sweden the authors suggest that childhood poverty is no longer binding on old age health once adult conditions are considered. This may be explained in two ways. The extensive welfare state of Sweden has been known to deliver exceptional health service to its older population. Our work comparing 17 countries in Europe also shows that Sweden has managed to break the link between economic position and sensory impairment^36^. Lennartsson and colleagues may have found a manifestation of Sweden’s exceptionalism in ameliorating pain, fatigue and breathing difficulty. But this can sit with the results above: on frailty childhood poverty continues to matter^5^. Sweden’s exceptionalism may not have succeeded in breaking all the links between childhood poverty and the spectrum of health in old age. If indeed the mechanism involves epigenetic changes, amelioration is possible but the chances of complete elimination are slim.

### Future research and policy including the UN Decade of Healthy Ageing

In England the Chief Medical Officer recently reported on critical plans to ensure healthy ageing in the country^10^. The report entitled "Health in an Ageing Society” emphasises within-country spatial disparity in frailty, in particular showing older adult with greater need of frailty treatment often reside in coastal and rural areas which in turn presents specific extra challenges. The report reproduced a map of spatial disparity in frailty which we drew to highlight this challenge that needs more investigation in any country^9^.

More broadly, the multi-country evidence presented here raises several implications for research and global health policy. First, retrospective childhood information can be more fruitfully examined by recognising that this information is laced with error. Childhood poverty, a latent construct indicated by several indicators of recalled information, can be obtained as a latent class^1–3^. Second, this strong result from many countries speaks directly to global health policy such as the UN Decade of Healthy Ageing. The initiative has missed an opportunity to gain control of the life course shaping of health in older ages around the world. If rich countries with their advanced health systems have experienced this life course shaping of health, the low and middle income countries are also likely to carry the long reach of childhood poverty. The 29 countries in this study are high income countries; those who grew rich before growing older. Other countries are in a decidedly more difficult predicament of growing old before growing rich e.g. India and China. The WHO observed that the countries with the difficult predicament are also growing old faster: what took France more than a century will take China only a few decades^37^. These countries should be the focus of future research on the life course shaping of health in older ages. With the higher prevalence of childhood poverty in low and middle income countries and its long arm reaching into old age as evinced here, the need for research in low and middle countries is urgent.

### Limitations and strengths

There are limits to what can be learned from this work. First, its framework is not a full structural framework^4^, although this has precedents^2,5,38^. That alternative has encouraged the authors to suggest that were more covariates considered, the association with childhood poverty would be entirely weakened. This suggestion is of course an eminently empirical question. The imperative remains that a fuller framework should be considered when information is available on youth and adult conditions in all these countries. Second, a key limitation arises from the design being observational rather than a randomised study (to childhood material intervention and to control), preventing causal interpretation of these results. The childhood poor may differ from the non-poor in ways not observed. Moreover, some of the childhood poor may not have survived to take part in these longitudinal ageing studies. Although this survivor effect may suggest a direction of bias, its magnitude is unknown. Last, as noted above the analytic sample here differs in many ways from those who declined to give childhood information. But across 29 countries these are not entirely systematic. Two strengths need emphasis here. First, following recent works, this study uses latent construct to address errors in retrospective studies^2,3^. Second, this is the first analysis to study 29 countries in the family of Health and Retirement Study, with a key exposure and an outcome that speak directly to the UN Decade of Healthy Ageing. The geographic reach of the sample as well as the variety of health systems it encompasses, together strengthen the generalisation of the results to other nations.

In conclusion, childhood poverty reaches long into old age, risking frailty in older ages. While more knowledge is needed, especially from low and middle income countries on how epigenetic changes operate, strong evidence is at hand to help secure a decade of healthy ageing by proving the necessity of eliminating child poverty.

## Data Availability

HRS, SHARE and ELSA are freely available to researchers. Access can be obtained after registration with respective repositories (HRS) https://hrs.isr.umich.edu, (SHARE) www.share-project.org, and (ELSA) www.elsa-project.ac.uk.
